# Correspondence on “The Stockholm Convention at a Crossroads: Questionable Nominations and Inadequate Compliance Threaten Its Acceptance and Utility”

**DOI:** 10.1021/acs.est.5c05961

**Published:** 2025-07-16

**Authors:** Martin Scheringer, Narain Ashta, Miriam L. Diamond, Juliane Glüge, Alexandra Harrington, Matthew MacLeod, Gabriel Sigmund, Noriyuki Suzuki, Hideshige Takada, Zhanyun Wang

**Affiliations:** † Institute of Biogeochemistry and Pollutant Dynamics, 27219 ETH Zürich, 8092 Zürich, Switzerland; ‡ Empa - Swiss Federal Laboratories for Materials Science and Technology, Laboratory for Air Pollution/Environmental Technology, 8600 Dübendorf, Switzerland; § 7938 University of Toronto, Toronto, ON M5S 1A1, Canada; ∥ 4396 Lancaster University, Lancaster LA1 4YN, United Kingdom; ⊥ Department of Environmental Science, 7675 Stockholm University, 10691 Stockholm, Sweden; # Environmental Technology, 4508 Wageningen University & Research, 6700 AA Wageningen, The Netherlands; 7 13585 National Institute for Environmental Studies, Tsukuba 305-8506, Ibaraki, Japan; 8 Tokyo University of Agriculture & Technology, Fuchu, Tokyo 183-8509, Japan; 9 Empa - Swiss Federal Laboratories for Materials Science and Technology, Technology and Society Laboratory, 9014 St. Gallen, Switzerland

Wania and McLachlan^1^ continue a discussion about the work of the Stockholm Convention (SC) on Persistent Organic Pollutants (POPs). This is useful because some of the issues raised surrounding the implementation of the SC warrant concerted action to safeguard the effectiveness of the SC. However, several of their statements are unsubstantiated or factually incorrect and could mislead the discussion about the future work of the SC. Here, we first respond to some of their general statements about the functioning of the SC and then to their specific criticism of the nomination of UV-328.

According to the authors, “a large increase in the number of chemicals that fulfill the screening requirements could lead to a considerable expansion in the number of chemicals being eligible for nomination and listing.” They see this as a problem, primarily because the capacity of countries to implement SC decisions may be exceeded. However, contrary to the authors’ insinuation that a high number of nominations is a problem, the nominations show that the SC works well and addresses exactly the concern for which it was established, namely to protect human health and the environment from POPs. If there is a concern about too high a burden from implementing the SC, the logical response is to strengthen the *implementation* process, not to throttle the *nomination* process.

Ironically, we believe that the author’s assertion that too many nominations could “jeopardize the acceptance of the convention” has the logic completely backward. The SC is an international treaty regime in which all State Parties, in their sovereign capacities, have the equal ability to make nominations. Indications that there may be too many nominations could be seen as a challenge to the foundational principle of sovereign equality in this context and potentially would undermine the faith of State Parties and stakeholders in the SC.

In addition, in their discussion of the responsibility of State Parties and industry for emissions of listed chemicals, the authors pose the question, “Is it possible to transfer the responsibility of not producing and releasing chemicals which exhibit the characteristics of POPs from states to emitters?” As previously noted, the SC is an international treaty and thus is governed by the rules of international law, particularly in terms of the sovereignty of States. It might be possible, but would be a difficult and lengthy process, for State Parties to propose an amendment that would encourage the involvement of industry at a deeper level. However, to comply with international law the legal obligations of the SC can bind only States. Increasing the involvement of industry and emitters could, however, be an activity undertaken through the terms of the SC relating to international cooperation.

The authors’ comments on the screening stage of the nomination process also follow faulty logic. According to the authors, the screening stage is obsolete now because many chemicals—in their opinion, too many chemicals—possess properties of POPs and the screening stage therefore seems to be ineffective. However, irrespective of the number of chemicals screened in or screened out, the screening stage is essential because it provides a well-defined and informative basis for the much more extensive next stage of the assessment (preparation of a risk profile). The screening stage does not need to “screen out” many nominations. On the contrary, if it served as an overly strict gatekeeper, it would inhibit the application of the SC as a “living” treaty, which the authors acknowledge is a crucial element for the SC. The fact that many chemicals in commerce may pass through the screening stage indicates that there are many chemicals that could be of concern. Accordingly, what is required is stronger coordinated action for the sound management of chemicals. Moreover, because of the substantial effort required to prepare a nomination (and subsequently draft the risk profile and the risk management evaluation), parties to the SC carefully examine the POP properties of a chemical before starting the nomination process. In this prescreening, aspects such as production volume and general relevance of a chemical are considered, and chemicals that are not relevant are not nominated. Thus, the process contains “checks and balances” to ensure its relevance and effectiveness.

The authors proceed in their Perspective to criticize the nomination of UV-328 based on flawed logic and factual inaccuracies. Their statement, “According to these arguments, any chemical found in floating debris can be argued to potentially undergo LRET [long-range environmental transport] in the oceans”, is factually correct but totally misses the point of LRET assessments under the SC: (i) In the same way as other acknowledged POPs (and, importantly, without any reference to, or consideration of, transport by plastic debris), UV-328 does fulfill the four criteria of POPs of persistence, bioaccumulation, toxicity, and LRET. Nonpersistent plastic additives such as *o-*phthalates (“any chemical”) would not be nominated as POP candidates just because they can travel with plastic debris, and this was pointed out clearly at the POPRC meetings discussing UV-328.^2^ (ii) If a chemical fulfills the criteria of POPs, there is no reason why transport with plastic debris should not be considered as a relevant mechanism of LRET. This is in line with the spirit and plain text of the SC, which says that LRET can be established by “Monitoring data showing that long-range environmental transport of the chemical, with the potential for transfer to a receiving environment, may have occurred via air, water or migratory species”. Plastic debris is transported via water; i.e., it is an environmental distribution of materials that have left the technosphere and can no longer be controlled by human action. Therefore, movement with plastic fully qualifies as environmental transport. The authors suggested a possibility that UV-328 in seabird lipid in remote areas originated not from long-range-transported plastics but from fishing gear derived from nearby activities. However, a recent study has demonstrated LRET of UV-328 by plastic resin pellets that are not discharged from fishing boats but from coastal industrial areas.^3^

Furthermore, the authors’ criticism that an exposure “outlier” was used to establish the occurrence of adverse effects as a consequence of LRET is factually wrong. First, it has been demonstrated that the presence of plastic debris at the remote Gough Island and Marion Island, and the resulting exposure of birds to plastic additives, actually are the result of LRET.^4^ Second, high concentrations of UV-328 in the seabirds at Gough Island and Marion Island are not an “outlier” but result from bioaccumulation in those birds that ingest large amounts of plastic debris. Concentrations of plastic additives are highly variable; high concentrations are observed in some plastics, while additives were not detected in others.^3^ As a result, birds that ingest a large number (>100) of plastic pieces bioaccumulate the additive, while those that ingest only few plastic pieces may accumulate no additive. Due to the inhomogeneous distribution of additives in plastics, sporadically high concentrations should not be ignored as “outliers” but should be treated as valid and relevant findings.

According to the authors, median levels of exposure should have been used, but that would be misleading and scientifically incorrect. In the context of the SC, the concern is about high values of a chemical occurring in remote regions. Given the limited scope of environmental monitoring of UV-328, the detection of some high values strongly indicates more widespread environmental contamination. As long as these high values are clearly caused by LRET, they are exactly what matters.

Regarding increases in nominations and regrettable substitutions, the authors correctly observe that “regulatory restriction can lead to regrettable substitutions.” However, regrettable substitutions can occur in any regulatory setting and this is not a logical reason for curtailing efforts to expand the SC nomination process. Instead, the focus should be on using capacity building and other elements of the SC to assist in the development and evaluation of potential substitutes in order to avoid regrettable substitution.

Our key message is to highlight the robustness of current scientific assessments under the SC, which should remain free from political influence and open to all sovereigns to make nominations based on sufficient data. The SC plays a crucial role in protecting societies from POPs as a group of particularly hazardous chemicals. We, like the authors, value the Stockholm Convention and want to see it succeed. We believe that this can be accomplished by encouraging the parties to significantly strengthen the SC’s implementation. The scientific community has a vital role in conducting research to address policy-relevant scientific questions^5^ and in supporting efforts to identify additional POPs.
